# The preparation of Fe_2_O_3_-ZSM-5 catalysts by metal-organic chemical vapour deposition method for catalytic wet peroxide oxidation of *m*-cresol

**DOI:** 10.1098/rsos.171731

**Published:** 2018-03-14

**Authors:** Yi Yang, Huiping Zhang, Ying Yan

**Affiliations:** School of Chemistry and Chemical Engineering, South China University of Technology, Guangzhou, Guangdong 510640, People's Republic of China

**Keywords:** metal-organic chemical vapour deposition, *m*-cresol, Fe_2_O_3_-ZSM-5, catalytic wet peroxide oxidation, kinetics

## Abstract

Fe_2_O_3_-ZSM-5 catalysts (0.6 wt% Fe load) prepared by metal-organic chemical vapour deposition (MOCVD) method were evaluated in the catalytic wet peroxide oxidation (CWPO) of *m*-cresol in a batch reactor. The catalysts have a good iron dispersion and small iron crystalline size, and exhibit high stability during reaction. In addition, the kinetics of the reaction were studied and the initial oxidation rate equation was given. Catalysts were first characterized by N_2_ adsorption–desorption isotherms, scanning electronic microscopy, energy-dispersive spectroscopy, X-ray diffraction and X-ray photoelectron spectroscopy. Results show that extra-framework Fe^3+^ species (presenting in the form of Fe_2_O_3_) are successfully loaded on ZSM-5 supports by MOCVD method. Performances of catalysts were tested and effects of different temperature, stirring rate, catalyst amount on hydrogen peroxide, *m*-cresol, total organic carbon (TOC) conversion and Fe leaching concentration were studied. Results reveal that catalytic activity increased with higher temperature, faster stirring rate and larger catalyst amount. In all circumstances, *m*-cresol conversion could reach 99% in 0.5–2.5 h, and the highest TOC removal (80.5%) is obtained after 3 h under conditions of 60°C, 400 r.p.m. and catalyst amount of 2.5 g l^−1^. The iron-leaching concentrations are less than 1.1 mg l^−1^ under all conditions. The initial oxidation rate equation $-r_{A0}=7.2\times 10^{11}{\text{e}}^{-81300/RT}C_{A}$ is obtained for *m*-cresol degradation with Fe_2_O_3_-ZSM-5 catalysts.

## 1. Introduction

Cresol isomers including *m*-, *o*- and *p*-cresol have been widely used in many industries such as pharmaceuticals, surfactants, herbicides, insecticides, preservatives and resins [1,2]. As a result, effluents discharged from these industries always contain high concentration of cresols and other phenolic compounds. Among these pollutants, *m*-cresol is regarded as a significant threat to the environment, especially to surface water, groundwater sources and other water resources [3–5] for its toxicity and refractory nature [6,7]. Cresols are classified as persistent, priority, toxic chemical by USEPA [8]. Among three isomers of cresol, *m*-cresol exhibits the most refractory property [9], because the presence of methyl group can stabilize the phenyl ring system through induction effect, which makes the cleavage of the CH–CH_3_ bond become more difficult [10–12]. Furthermore, during the oxidation procedure, oxidants will attack the *para*-position of cresol in priority, and the methyl on the *meta*-position will restrain this procedure due to the hindering effect. Therefore, a way to remove *m*-cresol effectively has currently become an environmental and economic problem urgent to solve [13].

Several techniques have been developed for cresol wastewater treatment. Biological degradation was extensively investigated by many other researchers [9,14–17]. Advantages of biological technique include low cost and a few by-products [18,19]. However, low removal rates, long retention time [3,14] and inefficiency in high pollutant concentration [20] make this method greatly limited in practice. Physical adsorption [21–23] is generally expensive. Chemical methods consist of thermal treatment [21,24,25] and electrochemical oxidation [26]. Both of them may result in the formation of more toxic intermediates and high running cost. The shortcomings of these traditional methods lead to the emergence of new effective and clean technologies for the elimination of cresol in wastewater.

Recently, advanced oxidation processes (AOPs) have attracted a lot of attention for their ability to mineralize target pollutants by generating hydroxyl radical (·OH) [27]. Catalytic wet peroxide oxidation (CWPO), using H_2_O_2_ to generate hydroxyl radical, is one of the AOPs and it is popular for its mild conditions and non-toxic products [28]. CWPO consists of homogeneous and heterogeneous processes. Homogeneous process, such as Fenton process, has been used in the quick removal of *m*-cresol [3], but drawbacks such as limited range of pH, catalyst separation and regeneration, the possibility of secondary contamination [29] still exist. These can be overcome by heterogeneous process where catalysts can be easily separated, regenerated and re-used [30]. Iron oxides [31,32] are commonly used active components in CWPO processes, for their low costs and user-friendliness.

Metal-organic chemical vapour deposition (MOCVD), in which metal species are deposited on supports through the gaseous metal precursor, has been widely used in the semiconductor [33]. Recently, it has been considered to be applied to the catalyst preparation, because MOCVD allows growing thin coating with a uniform thickness on some complex topographical surfaces at relatively low temperatures. Conventional heterogeneous catalyst preparation methods include impregnation [34], ion exchange [35] and co-precipitation [36]. As we know, the catalytic performance of the catalysts is strongly related to the structural characteristics of the components such as particle size, shape and distribution over the support. Impregnation method is user-friendly and cheap, but the active components cannot be distributed uniformly over the pores and they are easy to agglomerate, thus affecting the catalytic activity. High quality requirement of support makes ion exchange method expensive. Co-precipitation can fix the active components in the support, but it is hard to control the structure of the catalysts. In addition, these methods require various procedures which may greatly change the catalyst structure and reduce their activity [37]. As a one-step preparation method, MOCVD could develop stable and well-structured catalysts without these disadvantages. Catalysts prepared by MOCVD could have a better metal dispersion, because the diffusion of the metal substance in the vapour phase is faster than in the liquid phase [38]. What is more, strong interaction will exist between the support materials and metal components [39]. Also, the active species leaching of catalysts could be reduced by MOCVD method [40,41].

Because of these advantages, MOCVD is gradually used for the synthesis of nanometre materials, highly dispersed oxide and high purity catalyst [42]. Also, MOCVD is facile, direct, novel and inexpensive, and these properties gain more and more attention for it. The Pd/ZIF-8 catalysts were prepared by MOCVD method in Zhang's group, and Pd nanoparticles were uniformly dispersed on ZIF-8 [43]. Likewise, Hu's group [41] prepared Cu/MCM-41 catalysts by MOCVD method, and they found that the level of metal leaching, which is normally seen in the heterogeneous solid–liquid-phase reaction, can be substantially reduced. They also reported the effect of oxygen on binding metal catalyst onto porous solid substrate in the MOCVD process. Avril *et al.* [44] grew porous cerium oxide thin films on silicon substrate through MOCVD methods and they studied the influence of deposition parameters on the thin film morphology. Chu *et al.* [40] also prepared a copper containing activated carbon catalyst by MOCVD, which performed a good catalytic activity in the catalytic wet air oxidation of phenol.

Liu *et al.* [45] studied the degradation of *m*-cresol in a fixed bed reactor with α-Fe_2_O_3_/γ-Al_2_O_3_ catalysts prepared by incipient wetness impregnation method, but the kinetics of the reaction had not been intensively studied. Little literature reports the study of CWPO process of *m*-cresol. Also, catalysts used for *m*-cresol degradation prepared by MOCVD have not been reported yet. In this work, ZSM-5 was chosen as the support because of its high hydrothermal stability, fine lipophilicity, strong chemical resistance and good catalytic activity [46]. The aims of this work are to (i) prepare novel Fe_2_O_3_-ZSM-5 catalysts which have a good iron dispersion and small iron crystalline size by MOCVD method, (ii) test the catalytic activity of the prepared catalysts in decomposition of *m*-cresol by CWPO process (the effects of operating parameters including reaction temperature, stirring rate and amount of catalysts on the CWPO procedure are also discussed) and (iii) study the kinetics of the catalytic reaction and give the initial oxidation rate equation of the *m*-cresol CWPO reaction.

## 2. Experimental set-up

### 2.1. Materials

Commercial ZSM-5 zeolites (Si/Al = 80) were supplied by Nankai University Catalyst Factory. Ferric acetylacetonate was purchased from Aladdin Industrial Corporation. Hydrogen peroxide (H_2_O_2_, 30 wt% aqueous) was purchased from Jiangsu Qiangsheng Chemical Co., Ltd. *m*-cresol and manganese dioxide were purchased from Shanghai Qiangshun Chemical Reagent Factory. Sodium thiosulfate and potassium iodide were purchased from Tianjin Bodi chemical Co., Ltd. Deionized water was self-made and used in all experimental processes. All of the chemical reagents used in this study were of analytical grade.

### 2.2. Catalyst preparation

**Figure 1.**
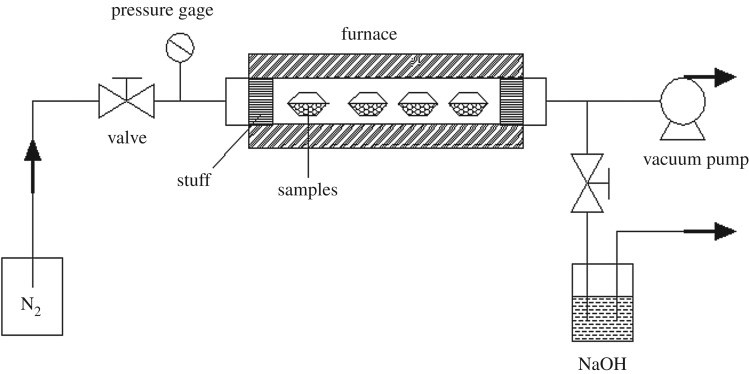
Schematic of the MOCVD system design.

The ZSM-5 zeolite columns were first smashed into powders and were divided into five groups (40, 60, 80, 100, 120 mesh) by the sieves. Fe_2_O_3_-ZSM-5 catalysts were prepared by MOCVD, and the schematic diagram is shown in figure 1. ZSM-5 and ferric acetylacetonate (Fe(acac)_3_) powders were chosen as support and iron source precursor [38], respectively. ZSM-5 supports were stored inside a crucible and well mixed with Fe(acac)_3_ precursor. The crucible was covered before it was put into a quartz tube. Nitrogen was used as protective gas during the process. The deposition temperature was 310°C with a heating rate of 5°C min^−1^ [47] and the deposition time was 1.5 h. After deposition, the catalysts were calcined in air at 550°C for 6 h with a heating rate of 1°C min^−1^. The iron load of the catalysts was 0.6 wt%.

### 2.3. Catalyst characterization

Different techniques were used to characterize the ZSM-5 and Fe_2_O_3_-ZSM-5 samples. N_2_ adsorption–desorption was performed on an ASAP2020 to get the isotherms, BET-specific surface area and pore volume. Scanning electronic microscopy (SEM) was performed on a Zeiss Merlin FE-SEM to obtain the textural and morphological information of the samples. Energy-dispersive spectroscopy (EDS) and element mapping were applied to analyse the iron dispersion on Fe_2_O_3_-ZSM-5 samples. X-ray diffraction (XRD) was performed on an X'Pert PRO instrument using Cu K*α* radiation (40 kV, 40 mA) with 2*θ* range from 5° to 90° to measure the crystallographic structures of the samples. X-ray photoelectron spectroscopy (XPS) spectra were tested on a Kratos Axis Ultra (DLD) instrument using an Al K*α* radiation source operated at 15 kV and 10 mA to obtain the binding energy of O_1s_ and Fe_2p_.

### 2.4. Catalytic wet peroxide oxidation of *m*-cresol

CWPO of *m*-cresol was performed in a glass batch reactor at atmospheric pressure for 3 h to test the catalytic activity of Fe_2_O_3_-ZSM-5 catalysts. During the reaction, the reactor was not sealed, and a condenser was used to prevent the loss of the liquid. The size of the catalysts was 178 µm (80 mesh). The batch reactor was heated by a thermal bath to set the desired temperature. A stirring rake was applied to mix the reactants well and eliminate the influence of external diffusion. The reaction solution contained 1 g l^−1^ *m*-cresol and 6 g l^−1^ H_2_O_2_. The amount of oxidant was adjusted to the stoichiometric amount needed for the total *m*-cresol oxidation according to the following reaction:

2.1 $${\text{C}}_{7}{\text{H}}_{8}\text{O}+17{\text{H}}_{2}{\text{O}}_{2}\to 7\text{C}{\text{O}}_{2}+21{\text{H}}_{2}\text{O.}$$

The required amount of H_2_O_2_ was first mixed with *m*-cresol and pre-heated to a desired temperature, and the catalysts were then added into the mixture. The reaction solution was sampled every 30 min to monitor the main parameters including conversions of *m*-cresol, H_2_O_2_, total organic carbon (TOC) and concentration of iron leaching. Reaction temperature, stirring rate and amount of catalysts were main variables in experiments and were changed systematically to study their effects on the reaction.

H_2_O_2_ concentrations were obtained by iodometric titration using 0.02 mol l^−1^ sodium thiosulfate solution and the expression of H_2_O_2_ conversion rate $\text{(}X_{{\text{H}}_{2}{\text{O}}_{2}})$ was as follows:

2.2 $$X_{{\text{H}}_{2}{\text{O}}_{2}}=\frac{C_{{\text{H}}_{2}{\text{O}}_{2}(0)}-C_{{\text{H}}_{2}{\text{O}}_{2}(t)}}{C_{{\text{H}}_{2}{\text{O}}_{2}(0)}}\times 100\%,$$

where $C_{{\text{H}}_{2}{\text{O}}_{2}(0)}$ and $C_{{\text{H}}_{2}{\text{O}}_{2}(t)}$ (mg l^−1^) are concentrations of H_2_O_2_ at the initial time *t* = 0 and sampling time *t* (hours), respectively.

After H_2_O_2_ concentrations were determined, 0.1 g manganese dioxide was added into sample solutions to eliminate the residual H_2_O_2_ [30] which may affect the measurement of other parameters. *m*-cresol concentrations were measured by applying high performance liquid chromatography (HPLC) (Agilent 1100) equipped with an Agilent HC-C18(2) column and a UV detector which set a wavelength of 272 nm. Methanol and ultrapure water (v/v = 80:20) were employed as the mobile phase and the flow rate was 1.0 ml min^−1^. The conversion rate of *m*-cresol $\text{(}X_{m\text{-cresol}})$ was calculated as follows:

2.3 $$X_{m\text{-cresol}}=\frac{C_{m\text{-cresol(0) }}-C_{m\text{-cresol(}t\text{) }}}{C_{m\text{-cresol}(0)}}\times 100\%,$$

where $C_{m\text{-cresol}(0)}$ and $C_{m\text{-cresol}(t)}$(mg l^−1^) are concentrations of *m*-cresol at the initial time *t* = 0 and sampling time *t* (hours), respectively.

TOC concentrations of samples were measured by a Liqui TOC instrument. The conversion rate of TOC $\text{(}X_{\text{TOC}})$ was as follows:

2.4 $$X_{\text{TOC}}=\frac{C_{\text{TOC}(0)}-C_{\text{TOC}(t)}}{C_{\text{TOC}(0)}}\times 100\%,$$

where $C_{\text{TOC(0) }}$ and $C_{\text{TOC(}t\text{) }}$ (mg l^−1^) are concentrations of *m*-cresol at the initial time *t* = 0 and sampling time *t* (hours), respectively.

Iron-leaching concentration in sampling solution was tested by atomic absorption spectrophotometer (AA-6800) to analyse the stability of the catalysts.

## 3. Results and discussion

### 3.1. Characterization

#### 3.1.1. N_2_ adsorption–desorption

**Figure 2.**
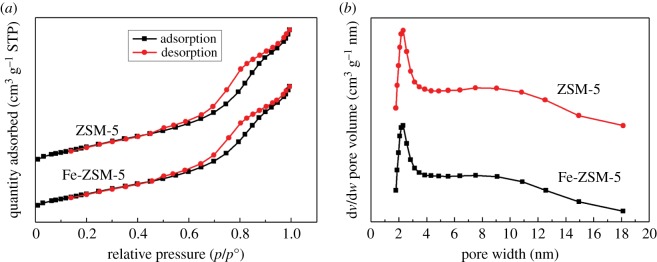
(*a*) N_2_ adsorption–desorption isotherms and (*b*) BJH pore diameter distributions of ZSM-5 support and Fe_2_O_3_-ZSM-5 catalysts.

N_2_ adsorption–desorption isotherms at 77 K of ZSM-5 support and Fe_2_O_3_-ZSM-5 catalyst are shown in figure 2*a*. Samples were degassed at 200°C in a vacuum for 6 h before measurement. The results are shown in table 1. The specific surface areas (*S*_BET_) are calculated from adsorption branches in the relative pressure range of 0.06–0.3 using the BET (Brunauer–Emmett–Teller) method. The total pore volume (*V*_total_) is calculated by analysing N_2_ adsorption–desorption isotherms. The microporous surface area (*S*_micro_) and microporous volume (*V*_micro_) are calculated using *t*-plot method. It can be seen that N_2_ adsorption–desorption isotherm shapes of ZSM-5 support and Fe_2_O_3_-ZSM-5 catalysts are similar to those of type II standard. In the beginning, the volume adsorbed increases continually with the increasing relative pressure, which should be attributed to monolayer–multilayer adsorption [48]. At a high relative pressure, both samples show a hysteresis loop and make the isotherm similar to type IV [49], implying the existence of mesopores which have narrowed size distribution on the samples [50]. The mesoporous pore size distributions are acquired by BJH (Barrett–Joyner–Halenda) method. As can be seen, a narrow peak can be observed between the pore width of 2 and 3 nm, indicating that mesopores between these sizes present a wide distribution in ZSM-5 support and Fe_2_O_3_-ZSM-5 catalyst [45]. The result matches well with the N_2_ adsorption–desorption isotherms.

**Table 1.** Physiochemical properties of the samples.

sample	*S*_BET_ (m^2^ g^−1^)	*S*_micro_ (m^2^ g^−1^)	*V*_micro_ (cm^3^ g^−1^)	*V*_total_ (cm^3^ g^−1^)
ZSM-5	276.2	124.1	0.060	0.343
Fe_2_O_3_-ZSM-5	251.7	109.3	0.053	0.313

As listed in table 1, after loading iron, the BET-specific surface area of ZSM-5 decreases from 276.2 to 251.7 m^2^ g^−1^ and the total pore volume also falls from 0.343 to 0.313 cm^3^ g^−1^. The results can be ascribed to the iron oxides which disperse in the pore canal and block the pores [51]. The microporous specific surface area and microporous volume also decrease by 14.8 m^2^ g^−1^ and 0.007 cm^3^ g^−1^, respectively, implying that the iron species are successfully loaded into the micropore and result in the loss of the microporous specific surface area and microporous volume.

#### 3.1.2. Scanning electronic microscopy and energy-dispersive spectroscopy analysis

**Figure 3.**
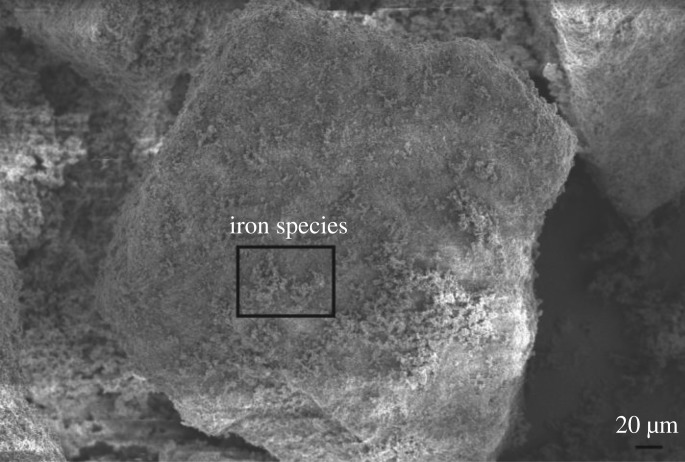
SEM image of Fe_2_O_3_-ZSM-5 catalysts.

**Figure 4.**
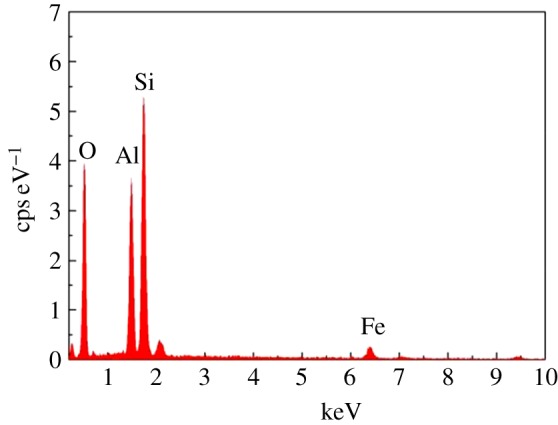
EDS elemental analysis spectrum of Fe_2_O_3_-ZSM-5 catalysts. cps, counts per second.

**Figure 5.**
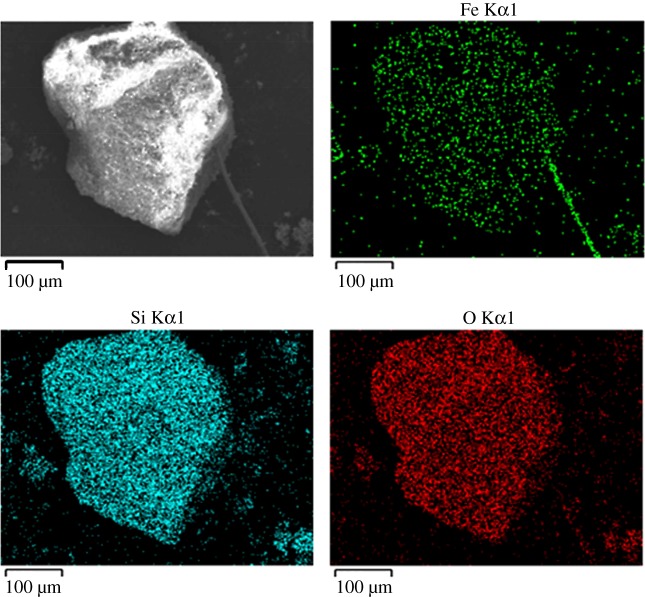
EDS elemental mapping images of Fe_2_O_3_-ZSM-5 catalysts.

Scanning electronic microscopy was used to study the morphology and structure of Fe_2_O_3_-ZSM-5 catalysts, and the image is shown in figure 3. As can be seen, iron species are observed and they are uniformly dispersed on the surface of the catalysts. The figures indicate that the iron species of our prepared Fe_2_O_3_-ZSM-5 catalysts are extra-framework Fe^3+^ species (Fe_2_O_3_), which are also located in the pores and on the surface of the zeolites. The results are essentially in agreement with EDS data exhibited in figures 4 and 5. In figure 4, the existence of Fe, Si, Al and O elements in catalysts is confirmed by the EDS elemental analysis spectrum. In figure 5, the distribution condition of different elements on the surface of catalysts is clearly depicted in the EDS elemental mapping images. It can be seen that Fe species are well dispersed on catalyst surface with a uniform dispersion, indicating that the active components are successfully loaded on the catalysts through MOCVD method.

#### 3.1.3. X-ray diffraction

**Figure 6.**
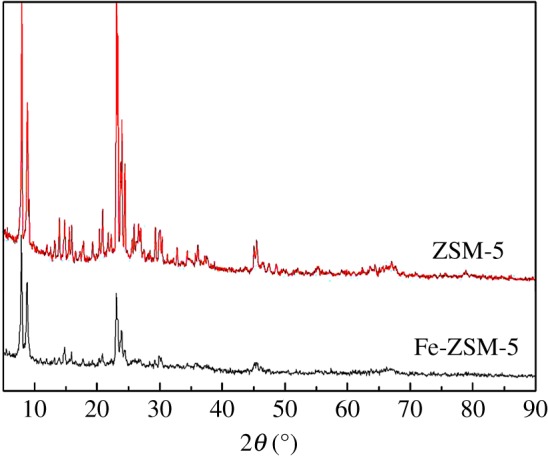
XRD patterns of ZSM-5 support and Fe_2_O_3_-ZSM-5 catalysts.

The XRD patterns of ZSM-5 support and Fe_2_O_3_-ZSM-5 catalyst are shown in figure 6. As can be seen, both samples give the diffraction peaks between 2*θ *= 7–9°and 2*θ* = 23–25°, matching well with the standard pattern of ZSM-5 according to the literature [52]. The patterns indicate that the crystal phase of ZSM-5 does not change after loading iron species. However, the intensity of characteristic ZSM-5 peak in Fe_2_O_3_-ZSM-5 catalyst decreases. According to the literature [48,53], the iron species loaded on ZSM-5 could enhance the zeolites' absorption of X-ray, causing the decrease in the intensity of characteristic ZSM-5 peaks. On the other hand, the characteristic peaks of Fe cannot be observed for the Fe_2_O_3_-ZSM-5 samples. Li *et al.* [54] have reported that the intensity of peak could be significantly affected by the size of the species and its dispersion on the support, while many other works [55–57] have proved that weak peaks in XRD patterns could be caused by good dispersion and small crystalline size of the component. According to Yoo [58], iron particles which were loaded on the support by CVD method would be small and would disperse well on the support. Zou *et al.* [56] also proved that no peak on the XRD pattern could be put down to fine dispersion of metal compounds on ZSM-5 support. Low content of the iron species might be another reason affecting the intensity of the peak. The existence of Fe_2_O_3_ on support can be proved by the following XPS analysis.

#### 3.1.4. X-ray photoelectron spectroscopy

**Figure 7.**
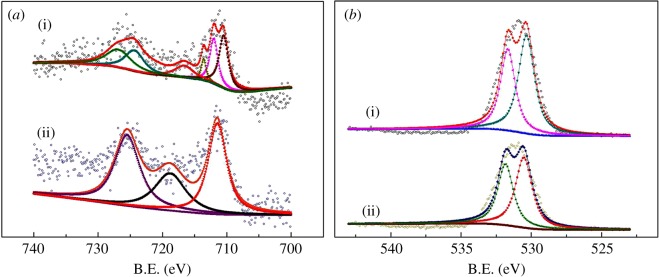
(*a*) Fe_2p_ XPS spectra for catalyst samples: (i) Fe_2_O_3_-ZSM-5 before calcination, (ii) Fe_2_O_3_-ZSM-5 after calcination. (*b*) O_1s_ XPS spectra for catalyst samples: (i) Fe_2_O_3_-ZSM-5 before calcination and (ii) Fe_2_O_3_-ZSM-5 after calcination. B.E., binding energy.

The results of XPS are displayed in figure 7. All spectra are calibrated by C_1s_ peak at 284.6 eV. As can be seen in figure 7*a*, the peaks of Fe_2_O_3_-ZSM-5 before calcination at 711.0 eV [59] and 726 eV [60] represent the binding energy of Fe_2p3/2_ and Fe_2p1/2_ in Fe(III), respectively. The satellite peak of Fe_2p3/2_ can be observed at 716.7 eV, which is the characteristic peak of Fe(III) in Fe_2_O_3_ according to many previous works [60–62]. In addition, the peaks centring at 722.6 eV and 709 eV represent the binding energy of Fe_2p3/2_ and Fe_2p1/2_ in Fe(Ⅱ), respectively, and the satellite peak of Fe_2p3/2_ locates at 714.6 eV, which can be attributed to Fe(Ⅱ) in FeO [60]. For Fe_2_O_3_-ZSM-5 after calcination, the spectrum is less complicated. The characteristic peaks of Fe(III) located at 711.0 eV, 726 eV and 718 eV are still obviously distinguishable, while the peaks of Fe(Ⅱ) disappear. The XPS results show that before calcination, different iron species with different iron valence including Fe(II) and Fe(III) are loaded on ZSM-5 support by MOCVD method, while after calcination, all species are oxidized to Fe(III).

The XPS spectra data of O_1s_ are shown in figure 7*b*. Shapes of XPS spectra for two samples are similar. The peak at 530.3 eV represents the characteristic of Fe–O bond. Another peak observed at 533 eV could be due to the dissociated oxygen in SiO_2_ [61] or hydroxyl groups absorbed on Fe_2_O_3_ [63].

### 3.2. CWPO of *m*-cresol over Fe_2_O_3_-ZSM-5 catalysts

#### 3.2.1. Effect of reaction temperature

**Figure 8.**
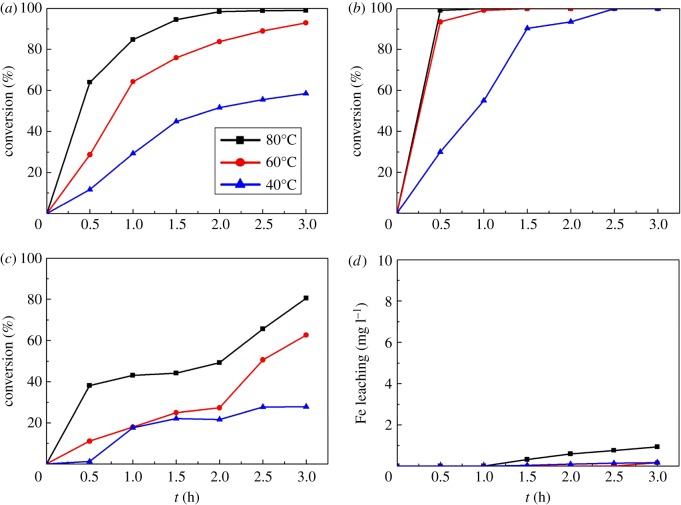
Effect of reaction temperature on catalytic performances (catalysts 2.5 g l^−1^, stirring rate of 400 r.p.m.): (*a*) H_2_O_2_ conversion, (*b*) *m*-cresol conversion, (*c*) TOC conversion and (*d*) Fe leaching concentration.

The CWPO reaction of *m*-cresol solution was carried out in a batch reactor at different temperatures (40°C, 60°C and 80°C). Other conditions were set as follows: catalysts 2.5 g l^−1^, stirring rate of 400 r.p.m. The performances of catalysts are shown in figure 8. In figure 8*a*, H_2_O_2_ conversion rises as the reaction temperature increases. The final H_2_O_2_ conversion of reaction temperature at 40°C, 60°C and 80°C reaches about 50%, 90% and 99%, respectively. The rise of H_2_O_2_ conversion can be ascribed to two reasons. The increasing temperature will promote the decomposition of H_2_O_2_ into hydroxyl radical [64] and accelerate the decomposition of H_2_O_2_ to H_2_O and O_2_. Figure 8*b* reveals that the conversion rate of *m*-cresol also increases as the reaction temperature increases. When the reaction temperature is 40°C, it takes 2.5 h for *m*-cresol conversion to reach 99%. When the temperature is increased to 60°C, it takes 1 h for 99% conversion. When the temperature is further increased to 80°C, it takes only 0.5 h. It has been reported that the degradation rate of organic pollutants in CWPO reaction can be enhanced by rising temperature [65], and this could be due to the production of more hydroxyl radicals which play an important part in the oxidation of *m*-cresol. We have known that the iron species of our Fe_2_O_3_-ZSM-5 catalysts are extra-framework Fe^3+^ species which present in the form of Fe_2_O_3_. According to Yan *et al.*'s [66] research, extra-framework Fe^3+^ species perform higher activity in organic degradation than the framework Fe^3+^, and this can be the reason of high *m*-cresol conversion in this work. The result is consistent with H_2_O_2_ conversion, implying that Fe_2_O_3_-ZSM-5 catalysts are active for *m*-cresol oxidation. A similar trend can also be observed in TOC conversion from figure 8*c*. It can be seen that elimination of TOC will be promoted as the temperature increases. For reaction temperatures at 40, 60 and 80°C, TOC conversion reaches 27.9%, 62.6% and 80.5%, respectively, after 3 h reaction. The residual TOC could still be detected while the *m*-cresol has been totally removed, indicating that *m*-cresol is degraded into some intermediate products and some hard degradable low weight molecular organic acids which produced the remainder TOC [67]. As for the stability of catalysts, iron-leaching concentration is shown in figure 8*d*. It can be observed that though the iron-leaching concentration gradually grows as the reaction temperature increases, the iron loss is low. When the temperature was 80°C, after 3 h, the iron concentration was 0.933 mg l^−1^, and only 6.2% of iron leached out from Fe_2_O_3_-ZSM-5. The loss of iron ions can be ascribed to the formation of the acid by-product during the reaction [68]. In addition, stirring for a long time could also wash out some of the active components. Even so, catalysts prepared by MOCVD still could significantly reduce the iron leaching, because the diffusion of the metal substance in vapour phase can help the metal components go further into the pore structure and finally be fixed in the pores, making the catalysts more stable [40].

#### 3.2.2. Effect of stirring rate

**Figure 9.**
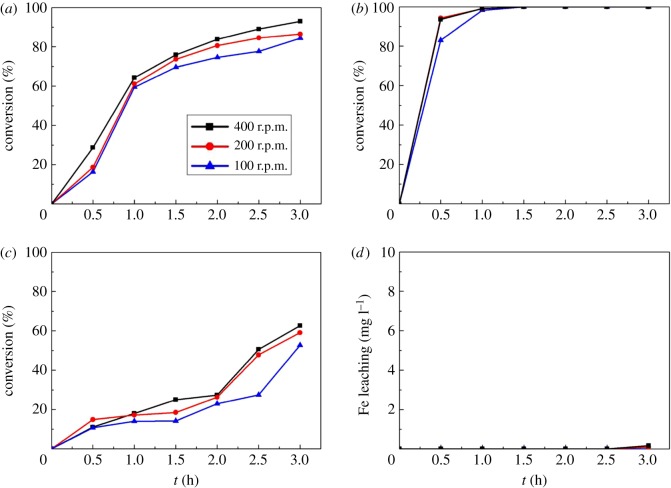
Effect of stirring rate on the catalytic performance (catalysts 2.5 g l^−1^, temperature at 60°C): (*a*) H_2_O_2_ conversion, (*b*) *m*-cresol conversion, (*c*) TOC conversion and (*d*) Fe leaching concentration.

Different stirring rate can affect the mechanical shearing force of catalysts, which will influence the loss of active component and the destruction of catalyst morphology. Stirring rate can also impact the mass transfer and external diffusion process, which play an important part in reaction rate, product conversion and selectivity. In this experiment, the stirring rate is adjusted to 100, 200 and 400 r.p.m. under same conditions (namely, catalysts 2.5 g l^−1^, temperature at 60°C) to study its impact on the reaction. The results are shown in figure 9. As figure 9*a* reveals, when the stirring rate increases from 100 to 400 r.p.m., H_2_O_2_ conversion is raised from 84% to 93%, implying that higher stirring rate will accelerate the decomposition of H_2_O_2_. As for *m*-cresol conversion depicted in figure 9*b*, when the stirring rate is 100 r.p.m., the conversion of *m*-cresol reaches only 83.0% after 0.5 h, and it takes 1.5 h for the conversion to reach 99%. When the stirring rate is raised to 200 and 400 r.p.m., only 1 h is needed to reach 99% conversion. The contact and mass transfer among reagents in the system are enhanced with the increasing stirring rate, thus promoting *m*-cresol degradation. By comparing figure 9*a* and *b*, the trend of *m*-cresol conversion is not synchronous with H_2_O_2_ conversion when the stirring rate increases, so it can be inferred that speeding up the stirring rate will not only promote the degradation of *m*-cresol but also boost the decomposition of H_2_O_2_ into oxygen and water. Figure 9*c* indicates that the variation trend of TOC conversion (52.7%, 59.0%, 62.6% at 100, 200, 400 r.p.m., respectively, after 3 h) is consistent with *m*-cresol conversion. When the stirring rate increases, TOC conversion simultaneous rises. At high stirring rate, external diffusion has been eliminated, so the degradation process of *m*-cresol is controlled by surface reaction on the catalysts and the reaction will not be significantly expedited as the stirring rate is further added. From figure 9*d*, we can see that though iron-leaching concentration ascends slightly with increasing stirring rate, the maximum concentration is only 0.164 mg l^−1^, and 1.1% of the iron is washed out into the solution, showing that the stirring rate does not have significant effect on the loss of active species. The result is consistent with the effect of temperature, indicating that the Fe_2_O_3_-ZSM-5 catalysts prepared by MOCVD method show good stability.

#### 3.2.3. Effect of amount of catalysts

**Figure 10.**
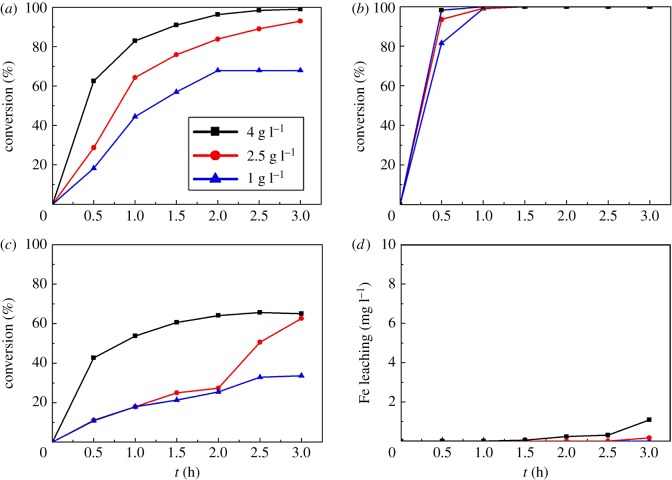
Effect of amount of catalysts on the catalytic performance (temperature at 60°C, stirring rate of 400 r.p.m.): (*a*) H_2_O_2_ conversion, (*b*) *m*-cresol conversion, (*c*) TOC conversion and (*d*) Fe leaching concentration.

To investigate the effect of catalyst amount on *m*-cresol degradation, different catalyst dosages (1, 2.5 and 4 g l^−1^) are added into the reactor under the following conditions: temperature at 60°C, stirring rate of 400 r.p.m. The results are shown in figure 10. As we can see in figure 10*a*, after 3 h reaction, conversion of H_2_O_2_ grows significantly from 67.9% to 99% as the catalyst amount increases from 1 to 4 g l^−1^. This phenomenon can be explained by Lewis's collision theory. The occurrence of a reaction needs to meet the requirement: two activated molecules collide in a particular direction (namely, effective collision). When more catalysts are added into the system, the number of active sites increases, so the effective collision probability between activated H_2_O_2_ molecules and active sites increases, and H_2_O_2_ decomposition is promoted. At the same time, formation of hydroxyl radicals is boosted and benefits the degradation of *m*-cresol. The result is corresponding to figure 10*b*. When the catalyst amount is 1 g l^−1^, conversion of *m*-cresol reaches 81.5% after 0.5 h. When the amount is adjusted to 2.5 g l^−1^, the figure obviously raises to 93.5%. When the dosage is 4 g l^−1^, 99% removal of *m*-cresol is observed. All three conditions get 99% removal after 1 h. As for TOC conversion portrayed in figure 10*c*, TOC has obvious higher conversion at condition of 4 g l^−1^ catalysts than at 2.5 and 1 g l^−1^. In the very beginning, conversion rates of 2.5 and 1 g l^−1^ are nearly the same. After 1.5 h, reaction at 2.5 g l^−1^ starts to exhibit higher TOC conversion than 1 g l^−1^. The results confirm that the increase of the catalyst dosages will take effect in *m*-cresol degradation. Iron-leaching concentration during reaction is depicted in figure 10*d*, and the highest value is 1.092 mg l^−1^. According to this figure, only 7.3 wt% of active component leaches out from the catalysts, and it is lower than catalysts prepared by incipient wetness impregnation [69]. According to Liu *et al.* [45], the leached iron ions have little effect on the TOC removal when the concentration is low. The result indicated that the catalysts prepared by MOCVD method exhibited excellent ability to degrade *m*-cresol with fine stability.

### 3.3. Kinetics of catalytic wet oxidation peroxide of *m*-cresol with Fe_2_O_3_-ZSM-5

**Figure 11.**
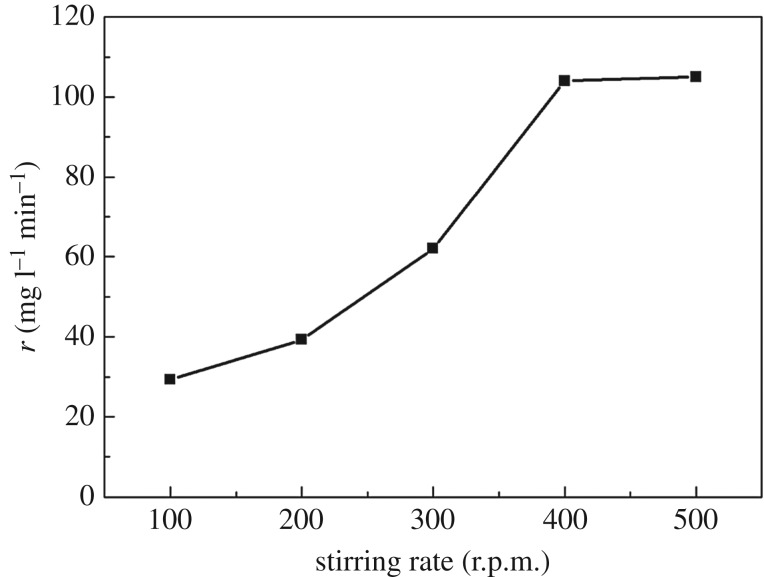
Effect of stirring rate on the initial rate of *m*-cresol conversion (catalysts 2.5 g l^−1^, H_2_O_2_ 9 g l^−1^, *m*-cresol 1 g l^−1^, temperature at 70°C).

**Figure 12.**
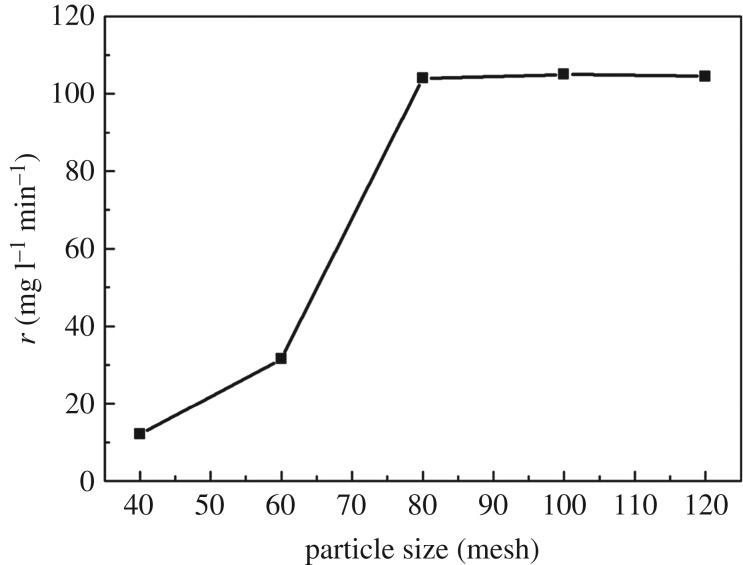
Effect of catalyst particle size on the initial rate of *m*-cresol conversion (catalysts 2.5 g l^−1^, H_2_O_2_ 9 g l^−1^, *m*-cresol 1 g l^−1^, temperature at 70°C, stirring rate of 400 r.p.m.).

To investigate the kinetics of CWPO of *m*-cresol, effects of stirring rate and particle size of catalysts on the initial rate of *m*-cresol conversion were studied first in order to eliminate the external diffusion and internal diffusion, respectively. The results are shown in figures 11 and 12. As can be seen, when the stirring rate is increased to more than 400 r.p.m., the initial rate of *m*-cresol degradation no longer rises. Similarly, when the particle size of catalysts gets to 80 meshes, the initial rate stops rising. The results mean that the external and internal diffusion have been eliminated at the stirring rate of 400 r.p.m. and particle size of 80 meshes.

If the CWPO of *m*-cresol over Fe_2_O_3_-ZSM-5 with excess H_2_O_2_ is a first-order reaction, it should follow the following reaction rate equation [70]:

3.1 $$-\frac{\text{d}C_{A}}{\text{d}t}=-r_{A}=k_{0}\text{exp}\;\left(-\frac{E_{a}}{RT}\right)C_{A},$$

where *C_A_* is *m*-cresol concentration (mg l^−1^) at time *t* (min), *k*_0_ (min^−1^) is the pre-exponential factor, *E_a_* (J mol^−1^) is the activation energy and *T* (Kelvin) is the reaction temperature. The equation could be changed into the following form:

3.2 $$\ln\frac{C_{A0}}{C_{A}}=kt.$$

**Figure 13.**
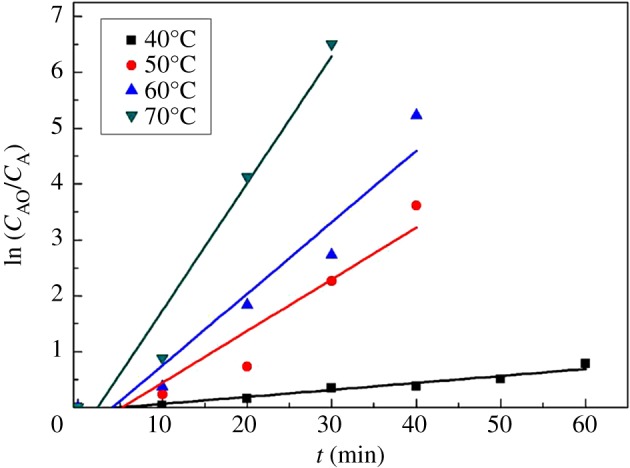
First-order oxidation of *m*-cresol by catalytic wet oxidation over Fe_2_O_3_-ZSM-5 (catalysts 2.5 g l^−1^, H_2_O_2_ 9 g l^−1^, *m*-cresol 1 g l^−1^, stirring rate of 400 r.p.m., particle size 80 meshes).

The first-order rate coefficient can be obtained by plotting ln(*C_A_*_0_/*C_A_*)versus time and the slope is the value of coefficient. The plots acquired at different temperatures from 313 to 343 K are shown in figure 13 (when the *m*-cresol conversion reached 100%, $\ln C_{A0}\text{/}C_{A}$ makes no sense, so these points are not depicted in the figure), and the values of the first-order rate coefficients are depicted in table 2. As can be seen, the experimental data are consistent with the rule of the first-order reaction, so it reveals that the catalytic reaction follows first-order kinetics.

**Table 2.** Kinetics constants for catalytic wet oxidation of *m*-cresol at different temperatures.

*T* (*K*)	*k* (min^−1^)	*R*^2^
313	0.0125	0.95
323	0.0927	0.91
333	0.1281	0.93
343	0.2275	0.96

According to Arrhenius equation:

3.3 $$k=k_{0}\text{exp}\;\left(-\frac{E_{a}}{RT}\right).$$

It can be changed into another form:

3.4 $$\text{ln}\;k=\text{ln}\;k_{0}-\frac{E_{a}}{R}\frac{1}{T}.$$

**Figure 14.**
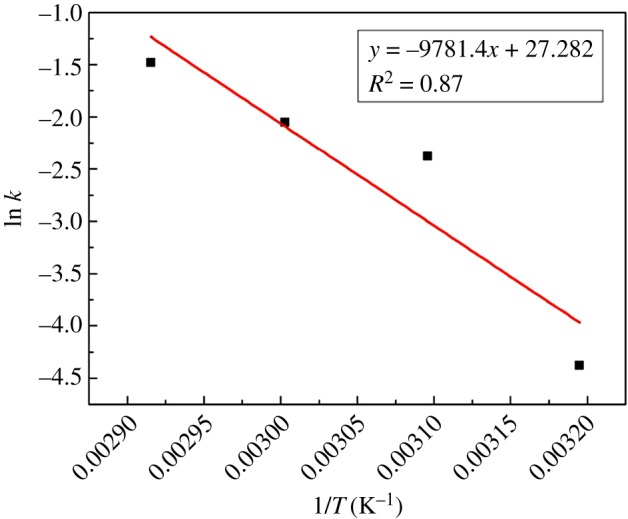
Arrhenius plot of ln *k* versus 1/*T*.

Arrhenius plot of ln *k* versus 1/*T* is depicted in figure 14. Based on the slope of the plot and the above equation, the activation energy is 81.3 kJ mol^−1^ and *k*_0_ is 7.2 × 10^11^ min^−1^. Hence, the initial oxidation rate could be expressed as follows:

3.5 $$-r_{A0}=7.2\times 10^{11}{\text{e}}^{-81300/RT}C_{A}.$$

## 4. Conclusion

Fe_2_O_3_-ZSM-5 catalysts with 0.6 wt% Fe load were prepared through MOCVD method and were tested in a batch reactor. The CWPO was carried out to investigate the effect of reaction temperature, stirring rate and the amount of catalysts on degradation of *m*-cresol. The performances of catalysts increase with higher temperature, faster stirring rate and larger catalyst amount in a certain range. Over 99% *m*-cresol removal and 80% TOC elimination are obtained under the conditions of 60°C, 400 r.p.m. and catalysts amount of 2.5 g l^−1^. Low Fe leaching (under 1.1 mg l^−1^) is detected under all conditions, implying that the catalysts exhibit high stability. The initial oxidation rate of *m*-cresol degradation with Fe_2_O_3_-ZSM-5 catalysts is $-r_{A0}=7.2\times 10^{11}{\text{e}}^{-81300/RT}C_{A}$. Results indicate that catalysts prepared by MOCVD show promising performances for *m*-cresol degradation and that can be a potential way to prepare catalysts for liquid-phase reaction like CWPO.

## Data accessibility

This article does not contain any additional data.
